# Starch depletion in the xylem and phloem ray parenchyma of grapevine stems under drought

**DOI:** 10.1093/aobpla/plad062

**Published:** 2023-08-30

**Authors:** Kyra A Prats, Ana C Fanton, Craig R Brodersen, Morgan E Furze

**Affiliations:** Department of Botany and Plant Pathology, Purdue University, 915 Mitch Daniels Blvd, West Lafayette, IN 47907, USA; Center for Plant Biology, Purdue University, 915 Mitch Daniels Blvd, West Lafayette, IN 47907, USA; Ecophysiologie et Génomique Fonctionnelle de la Vigne, INRAE, 210 Chemin de Leysotte, Villenave-d’Ornon 33140, France; School of the Environment, Yale University, 195 Prospect St, New Haven, CT 06511, USA; Department of Botany and Plant Pathology, Purdue University, 915 Mitch Daniels Blvd, West Lafayette, IN 47907, USA; Center for Plant Biology, Purdue University, 915 Mitch Daniels Blvd, West Lafayette, IN 47907, USA; Department of Forestry and Natural Resources, Purdue University, 715 Mitch Daniels Blvd, West Lafayette, IN 47907, USA

**Keywords:** Drought, nonstructural carbohydrates, phloem, ray parenchyma, xylem

## Abstract

While nonstructural carbohydrate (NSC) storage can support long-lived woody plants during abiotic stress, the timing and extent of their use are less understood, as are the thresholds for cell mortality as NSCs and water supplies are consumed. Here, we combine physiological and imaging tools to study the response of *Vitis riparia* to a 6-week experimental drought. We focused on the spatial and temporal dynamics of starch consumption and cell viability in the xylem and phloem of the stem. Starch dynamics were further corroborated with enzymatic starch digestion and X-ray microcomputed tomography imaging. Starch depletion in the stems of droughted plants was detected after 2 weeks and continued over time. We observed distinct differences in starch content and cell viability in the xylem and phloem. By the end of the drought, nearly all the starch was consumed in the phloem ray parenchyma (98 % decrease), and there were almost no metabolically active cells in the phloem. In contrast, less starch was consumed in the xylem ray parenchyma (30 % decrease), and metabolically active cells remained in the ray and vessel-associated parenchyma in the xylem. Our data suggest that the higher proportion of living cells in the phloem and cambium, combined with smaller potential NSC storage area, rapidly depleted starch, which led to cell death. In contrast, the larger cross-sectional area of the xylem ray parenchyma with higher NSC storage and lower metabolically active cell populations depleted starch at a slower pace. Why NSC source-sink relationships between xylem and phloem do not allow for a more uniform depletion of starch in ray parenchyma over time is unclear. Our data help to pinpoint the proximate and ultimate causes of plant death during prolonged drought exposure and highlight the need to consider the influence of within-organ starch dynamics and cell mortality on abiotic stress response.

## Teaser

We showed that starch dynamics and cell viability differed between the xylem and phloem in the stem of *Vitis riparia* throughout an experimental drought. While starch and living cells were absent in the phloem following the drought period, starch and a few populations of living cells remained in the xylem.

## Introduction

Long-lived woody plants face abiotic stress that has the potential to compromise plant organs and overall plant health. The ability to withstand abiotic stress is thus essential for the long-term survival of perennials and is in part governed by carbon (C) storage and allocation processes (Hartmann *et al.* 2020). For example, drought typically triggers stomatal closure (Brodribb and Holbrook 2003; Brodribb and McAdam 2017), which affects both the xylem and the phloem: water transport through the xylem is significantly reduced, the production of sugars and their export from the leaves to the phloem is also reduced, and stored nonstructural carbohydrates (NSCs) may begin to be drawn upon to support respiration (McDowell *et al.* 2008, 2011; Sala *et al.* 2010). The disruption of these interconnected systems has been widely recognized to lead to plant mortality (McDowell *et al.* 2008). While there has been extensive research documenting how drought impacts the response and recovery of xylem water transport, such as the extent and spread of embolism in the stem during drought (McDowell *et al.* 2008; Brodribb and Cochard 2009; Brodersen and McElrone 2013; Urli *et al.* 2013; Choat *et al.* 2018), a spatial assessment of within-organ NSC dynamics is critically needed to provide a more comprehensive picture of how individual organs influence the whole-plant response to drought.

Nonstructural carbohydrates, composed primarily of soluble sugars and insoluble starch, are the products of photosynthesis and are used by plants across timescales; while sugars often support more immediate functions involving transport, metabolism and osmoregulation, starch can be stored in parenchyma cells for later use (Hartmann and Trumbore 2016; Hartmann *et al.* 2020). Stored starch reserves serve as a potential resiliency mechanism because they can be remobilized for use under C limiting stress (e.g. drought). The effect of drought on NSCs has been mixed; NSCs have been shown to increase, decrease or are maintained at pre-drought concentrations. The highly variable response of NSCs to drought is likely due to a myriad of factors, including differences between species, organs, ontogeny, legacy effects of previous stress and environmental conditions (Adams *et al.* 2017). Nevertheless, NSC depletion has been commonly observed in woody perennial plants in response to drought. For example, total NSCs and sugars declined in the stem of mature temperate trees following natural drought (Blumstein and Furze 2022). In *Vitis vinifera*, NSCs—particularly starch—also declined under experimental water stress in the leaves of cv. Barbera (Tombesi *et al.* 2022) and in the stems of cv. Sangiovese (Vuerich *et al.* 2023), while glucose concentrations declined in the petioles of cv. Syrah and cv. Cabernet sauvignon (Falchi *et al.* 2020).In a recent review, 63 % of temperate and boreal angiosperm species and 67 % of gymnosperm species had decreased NSC concentrations in at least one organ at the time of drought-induced mortality (Adams *et al.* 2017).

However, our current understanding of the dynamic processes of NSC storage and consumption during drought is incomplete. Existing methods to determine NSC concentrations in organs are typically destructive, and provide a concentration on a mass or volume basis (Landhäusser *et al.* 2018), where the spatial distribution of starch within the sample is lost. Thus, destructive organ-level measurements fail to capture the finer-scale inter- and intra-tissue C dynamics. For example, the stem is a major NSC storage organ, and stem NSCs are stored within parenchyma, with ray parenchyma spanning the phloem, cambium and xylem and potentially forming a continuous tissue that extends deep into the sapwood and heartwood (Furze *et al.* 2018). The metabolic activity of these tissues along with the distribution and accessibility of the stored NSCs within them could influence the physiological mechanisms underlying responses to abiotic stress. Recent work using X-ray microcomputed tomography (microCT) imaging showed the depletion of starch in the xylem ray parenchyma of grapevine stems in response to shade (Earles *et al.* 2018) and set the precedent for understanding spatial and temporal starch dynamics in both the xylem and phloem in response to other abiotic stressors like drought.

Here, we exposed the ecologically and economically important woody perennial *Vitis riparia* (Riverbank grape) to a 6-week experimental drought and used a suite of staining and imaging methods to sequentially visualize the temporal and spatial distribution of starch in the stem to determine when and where starch depletion within the ray parenchyma occurred, as well as the coincidence of starch depletion with cell mortality. Our main objective was to visualize starch content and cell viability in both the xylem and phloem as a function of drought duration. These data were complemented by physiological measurements (e.g. continuous stem water potential, photosynthetic rate, chlorophyll fluorescence and enzymatically derived starch concentration) and supported by microCT imaging of stems. We hypothesized that starch depletion in the ray parenchyma would occur in plants exposed to drought compared to well-watered control plants, and that starch depletion would continue throughout the drought (i.e. amount of starch at 2 weeks > 4 weeks > 6 weeks). Importantly, we predicted that the timing and extent of starch depletion in the ray parenchyma would differ between the xylem and phloem. In trees, NSCs have been shown to be highly dynamic and accessible in the outer portions of the stem near the phloem and active xylem sapwood (Richardson *et al.* 2015; Furze *et al.* 2020), so we expected that starch depletion would occur in the phloem ray parenchyma first and would continue to deplete radially into the xylem ray parenchyma towards the centre of the stem as the drought went on. Overall, by investigating drought-induced physiological changes within xylem and phloem tissues, our work advances our knowledge of the role within-organ C dynamics play in abiotic stress response.

## Materials and Methods

### Plant material and growth conditions

*Vitis riparia* plants (Riverbank grape; 1-year old, grade one, own-rooted; *n* = 12) were received from Double A Vineyards (Fredonia, NY, USA) in April 2020. This species is native to more mesic habitats of eastern North America and encompasses a large latitudinal range (USDA and NRCS 2023). A previous water relations study conducted on this species in a common garden vineyard experiment in California classified *V. riparia* as drought-sensitive; this classification was based on drought-induced reductions in net assimilation rate, stomatal conductance and other water relations parameters compared to well-watered controls and after comparison with other *Vitis* species (Padgett-Johnson *et al.* 2003).

Plants were potted in 10 L plastic pots filled with a peat-based growing medium containing perlite, vermiculite and slow-release fertilizer (Premier Pro-Mix BX, Premier Horticulture Inc.). Growth was maintained under greenhouse conditions (day/night temperature of 35 ± 8/25 ± 4 °C, photoperiod of ~12.5 h, and relative humidity of 57 ± 15 %). Plants were trained to have a primary trunk (herein referred to as stem) by initially pruning lateral growth and were drip-irrigated two times daily for 10 min each watering time (0.66 L per day) prior to the start of the experimental drought.

### Baseline physiological measurements

Three weeks prior to the start of the drought experiment, we collected weekly physiological measurements. All measurements were taken between approximately 11:00 and 14:00. Soil moisture (volumetric water content, %) was measured in three locations for each pot using a ThetaProbe type ML2x sensor connected to a HH2 Moisture Meter (Delta-T Devices Ltd, Cambridge, UK). Photosynthesis and chlorophyll fluorescence were measured on one leaf per plant using a LI-6400 equipped with a leaf chamber fluorometer (6400-40, Li-Cor, Lincoln, NE, USA). Photosynthetic assimilation was measured with conditions set to 500 µmol m^−2^ s^−1^ photosynthetic photon flux density, 400 ppm CO_2_ and 200 μmol s^−1^ airflow and measurements were logged three times after values stabilized. To measure chlorophyll fluorescence, leaves were dark-adapted for 1 h using dark-adapting clips (9964-091, Li-Cor, Lincoln, NE, USA). The leaf chamber fluorometer was then used to measure the minimum value of chlorophyll fluorescence in the dark-adapted state (*F*_o_) prior to applying a saturating pulse of light to the leaf to induce and measure the maximum value of fluorescence in the dark-adapted state (*F*_m_). The difference between *F*_o_ and *F*_m_ is the variable fluorescence *F*_v_; *F*_v_/*F*_m_ was determined and indicated the maximum quantum efficiency of Photosystem II (Murchie and Lawson 2013).

Stem water potential was measured for each plant using a Scholander pressure chamber (Model 600, PMS Instruments, Albany, OR, USA). Leaves were covered and sealed with mylar bags for 30 min prior to measurement to bring the leaves into hydraulic equilibrium with stem water potential. Each leaf was then excised at the base of the petiole with a razor blade and placed into the pressure chamber with the cut end protruding from the sealed chamber. The chamber was pressurized and the pressure at which water started to emerge from the cut end was recorded and defined as stem water potential.

### Drought treatment and monitoring

To induce water stress by drought, irrigation was removed on 26 August 2020 following morning watering. The plants were randomly assigned to four treatment groups: well-watered control (*n* = 3), 2-week drought (*n* = 3), 4-week drought (*n* = 3) and 6-week drought (*n* = 3). The treatment name indicates the duration of drought exposure prior to tissue sample harvest for each group (e.g. the 2-week drought group was harvested after 2 weeks). The same baseline measurements (soil moisture, photosynthesis, chlorophyll fluorescence and stem water potential) were collected weekly throughout the drought experiment between approximately 11:00 and 13:00. Photosynthesis and chlorophyll fluorescence measurements were not measured for droughted plants in the final 2 weeks (weeks 5 and 6) of the experiment because they no longer had healthy leaves [**see**Supporting Information—**Fig. S1**].

For a subset of plants, we also continuously monitored stem water potential throughout the experimental drought with stem psychrometers (PSY1 Stem Psychrometer, ICT International, Armidale, NSW). One plant per treatment group was outfitted with a psychrometer installed on the stem between the first and second nodes, approximately 5 cm above the soil. To ensure that the psychrometers were giving valid measurements of stem water potential, we compared stem water potential measured by each of the four psychrometers with pressure chamber measurements on leaves the day prior to the start of the experimental drought [**see****Supporting Information—Fig. S2**]. The psychrometers needed to be reinstalled every 2 weeks due to plant growth and wound responses at the installation site. Rather than uninstalling and reinstalling the psychrometers onto the same plants, which would risk girdling them due to the small stem diameter, the psychrometers were reinstalled onto a different plant within the same treatment each time.

### Sequential harvesting of plants

After 2 weeks of drought, plants in the 2-week drought group (*n* = 3) were harvested. Similarly, plants in the 4-week drought group (*n* = 3) were harvested after 4 weeks of drought, and so on for the 6-week drought group (*n* = 3). Plants in the well-watered control group (*n* = 3) were also harvested after 6 weeks to conclude the experiment.

For each harvest, plants were removed from the greenhouse and brought into the laboratory. The height of each plant was measured and stem material was collected from two points along the stem, 10 and 50 % of the total plant length above the soil surface. Based on evidence that different trunk locations of large, mature tree stems have similar NSC concentrations (Richardson *et al.* 2015), we expected that starch concentrations would also be similar along the length of the stem given the smaller height and stem diameter of grapevines. However, we still sampled two stem locations to ensure that the response to drought did not differ along the length of the stem. The 50 % height was selected given its intermediate location; 10 % was selected to capture any influence of the root system on stem starch dynamics and was also the closest location to the soil surface that avoided psychrometer installation. Stem material from both locations was immediately used for sectioning and staining to visualize and quantify starch and viable/metabolically active cells. Additionally, the stem (ca. 4 cm length, 10 % above the soil) was sampled at the time of harvest and divided for enzymatic measurement of starch and microCT imaging.

### Quantifying starch in xylem and phloem ray parenchyma of the stem with iodine staining

To visualize and quantify starch in the xylem and phloem ray parenchyma of the stem, the stem material collected during each harvest was immediately sectioned using a microtome (GLS1 microtome, Schenkung Dapples, Zurich, Switzerland) to produce 10 μm thick cross-sections of the stem at two locations, 10 and 50 % of the total plant length above the soil surface. Following the method of Fanton *et al.* (2022), the sections were immediately submerged in iodine solution for 60 s to stain starch granules, rinsed with water, mounted on glass slides and imaged with a camera (Canon 6D DSLR, Melville, NY, USA) and compound microscope (Olympus BX40, Olympus America, Center Valley, PA, USA). The portion of the stem imaged was representative of the presence and spatial distribution of starch within the xylem and phloem ray parenchyma of the entire cross-section. The iodine solution was prepared by diluting (1:5 volume) 2 % iodine tincture USP (Swan, Vi-Jon, Inc., St. Louis, MO, USA) in water.

Using these images, we first confirmed that the potential storage area (i.e. ray parenchyma in the xylem, phloem and in total) did not differ between plants in each drought group. Then the percent of starch in the xylem ray parenchyma and the percent of starch in the phloem ray parenchyma was quantified using ImageJ (version 1.53) (Abràmoff *et al.* 2004). For each image, we traced the xylem ray parenchyma and phloem ray parenchyma independently and then converted the image to an 8-bit binary image. The xylem ray parenchyma was selected and cropped and then we used the thresholding tool to identify starch in the xylem ray parenchyma. The thresholding tool works by identifying pixels between two values and colouring them with a threshold colour. The value range is determined by manually adjusting the minimum and maximum values until the threshold colour overlays the area of interest (i.e. starch). The same threshold range (minimum gray value 0, maximum gray value 60) was applied to the xylem ray parenchyma because it aligned well with starch for all images. This process was then repeated for the phloem ray parenchyma. However, to more accurately identify starch within the phloem ray parenchyma, we needed to adjust the threshold range for each image (minimum gray value 0 for all images, maximum gray value 60 for 63 % of images, 70 for 12 % of images and 120 for 25 % of images). We then calculated the area of the threshold starch in the xylem ray parenchyma, the area of the xylem ray parenchyma, the area of the threshold starch in the phloem ray parenchyma and the area of the phloem ray parenchyma. These values allowed us to calculate the percent of starch in the xylem ray parenchyma ((area of threshold starch in xylem ray parenchyma/area of xylem ray parenchyma) × 100), the percent of starch in the phloem ray parenchyma ((area of threshold starch in phloem ray parenchyma/area of phloem ray parenchyma) × 100), and the percent of starch in the whole rays (((area of threshold starch in xylem ray parenchyma + area of threshold starch in phloem ray parenchyma)/(area of xylem ray parenchyma + area of phloem ray parenchyma)) × 100). Percent starch data are provided in **Supporting Information—Table S1** and data for both stem locations are displayed in **Supporting Information—Fig. S3**.

### Quantifying starch concentrations in the stem with enzymatic digestion

Stem material (ca. 3 cm length, 10 % of the total plant length above the soil) was used for starch analysis. Samples were immediately placed in a drying oven at 100 °C for 1 h to deactivate starch-degrading enzymes, and then at 70 °C for 2–3 days until they were completely dry. The dried samples were ground (Thomas Scientific Wiley Mill, Swedesboro, NJ, USA, mesh 40) and 30 mg of each sample was weighed out for starch analysis. This allowed us to obtain starch concentrations (mg g^−1^) that could be compared to the starch patterns obtained by iodine staining. Following the standard protocol by Landhäusser *et al*. (2018), ground material was sequentially digested with α-amylase and amyloglucosidase to produce glucose hydrolysate (Landhäusser *et al.* 2018). A peroxidase-glucose oxidase colour reagent was applied and samples were measured at 525 nm (Ocean Insights, USB4000 Fiber Optic Spectrometer). Starch concentrations are provided in **Supporting Information—Table S1**.

### Visualizing starch in the stem using X-ray microCT

As qualitative support for our findings from the iodine staining method, we also used microCT imaging to visualize starch. An additional segment (ca. 1 cm length, 10 % of the total plant length above the soil) from each dried stem was collected and scanned at the microCT facility (Beamline 8.3.2) at the Lawrence Berkeley National Laboratory Advanced Light Source (ALS) with a 3.25-µm pixel resolution. Samples were scanned at 24 keV around 180° in continuous tomography mode, which produced two-dimensional projection images. Stems were dried using the same drying procedure used to prepare stem samples for enzymatic starch digestion, above. Dried stems were imaged instead of intact living plants due to restricted facility access imposed by the pandemic. The raw data were reconstructed into a three-dimensional dataset using a custom python script (Gürsoy *et al.* 2014). Transverse images from the microCT image datasets were visualized in ImageJ to see the distribution of starch (Earles *et al.* 2018) for all treatment groups [**see****Supporting Information—Fig. S4**].

### Visualizing cell viability and metabolic activity in the stem with fluorescein diacetate staining

To visualize cell viability in the xylem and phloem, we made 20-μm cross-sections from both stem locations and stained them (green signal) with the cell viability assay stain fluorescein diacetate (FDA) (Knipfer *et al.* 2016). Fluorescein diacetate has been used as a quick and reliable method for detecting viable plant cells (Widholm 1972). Nonpolar FDA is incorporated into viable cells, where it is hydrolysed by esterases to produce fluorescein; fluorescein stains the cytoplasm green (Saruyama *et al.* 2013). Following Knipfer *et al.* (2016), a 9.6 μM FDA solution (Sigma-Aldrich, Milwaukee, WI, USA) was prepared by adding 2 μL of 4.8 mM stock FDA solution (in acetone) to 1 mL of water (Knipfer *et al.* 2016). Sections were immediately submerged in the FDA solution and incubated in the dark for 30 min. Each section was then rinsed with water, mounted on a glass slide and imaged under blue light (cube U-MNIB, excitation filter 470–490 nm, dichroic mirror 505 nm, barrier filter 515IF--) using a fluorescence microscope (Olympus BX60). At the same time, a comparable set of sections were submerged in water instead of FDA solution and was imaged to account for autofluorescence. To provide a quantitative assessment of fluorescence in FDA-stained stem images, fluorescence intensity was measured using FIJI (version 2.14.0/1.54f) and displayed in **Supporting Information—Fig. S5**. For each RGB image, the green colour channel was extracted and an identical rectangular region of interest was placed in the phloem, xylem and image background. Fluorescence intensity was determined for the xylem and phloem by measuring the integrated density in each region of interest and subtracting the background signal.

### Statistical analyses

**Figure 1.**
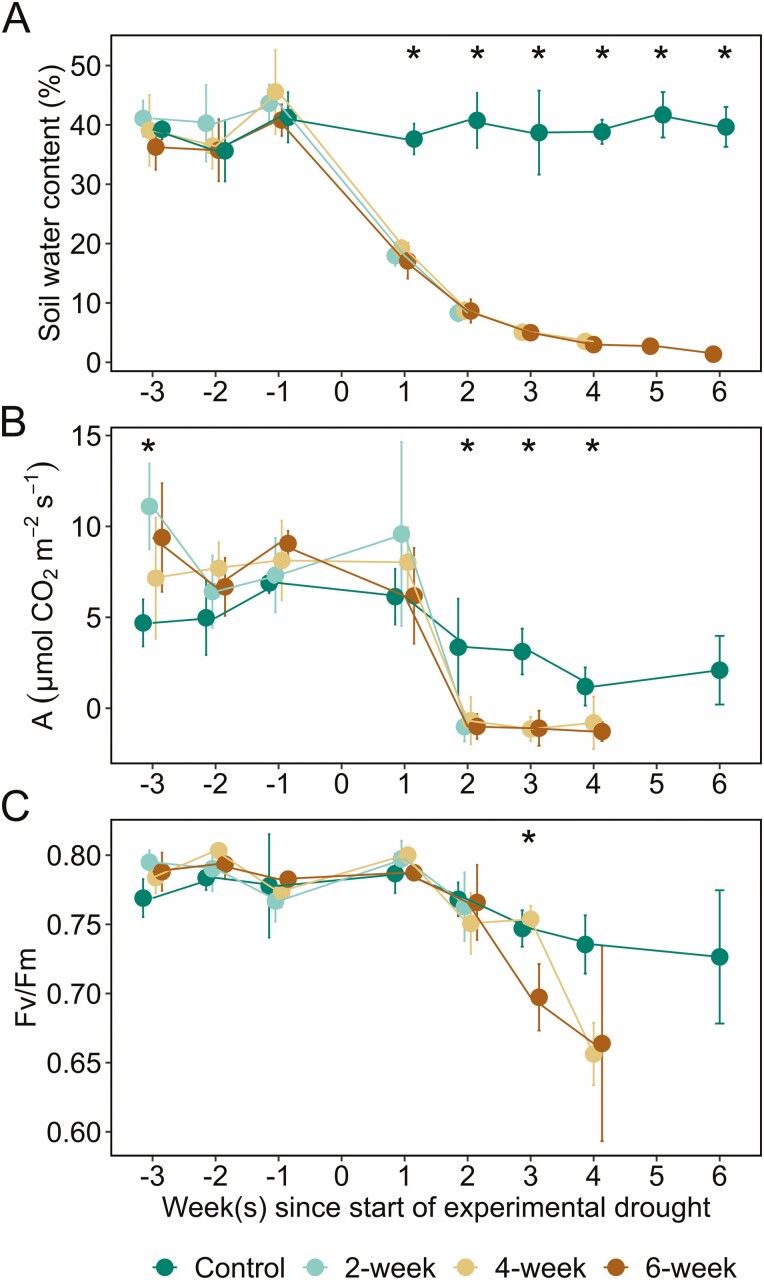
Environmental and physiological parameters measured before and during the experimental drought for four treatment groups. The parameters measured were (A) soil water content, (B) assimilation (*A*), and (C) chlorophyll fluorescence (*F*_v_/*F*_m_). Zero marks the start of the experimental drought. Measurements were collected from all treatments for three weeks leading up to the start of the drought and then each group was measured for 2, 4 or 6 weeks of drought, respectively, with the well-watered control group measured for 6 weeks. The lack of data for the 6-week drought group beyond week 4 in (B) and (C) was due to the plants no longer having healthy leaves for measurement [**see****Supporting Information—Fig. S1**]. Error bars denote ± 1 SD of the mean. An asterisk indicates treatment groups significantly differed from each other at a given timepoint based on Tukey’s honest significant difference (HSD) at *α* = 0.05.

To compare physiological traits between treatment groups, we used a one-way ANOVA to analyse each physiological variable among treatments at each time point (Fig. 1). The physiological variables compared among treatments were soil moisture (soil water content; Fig. 1A), photosynthetic rate (*A*; Fig. 1B) and chlorophyll fluorescence (*F*_v_/*F*_m_; Fig. 1C). Photosynthesis measurements were recorded three times for each plant and soil moisture was measured in three locations for each plant, so a nested one-way ANOVA was used in both cases. For significant ANOVAs, differences between pairs of means were evaluated with Tukey’s honest significant difference (HSD), *α* = 0.05.

We first checked to see if percent starch differed between stem locations by performing a two-way ANOVA to analyse the percent starch in the xylem ray parenchyma among treatments and stem locations (treatment × stem location; **Supporting Information—Fig. S3A**). The same analysis was repeated for both phloem ray parenchyma [**see****Supporting Information—Fig. S3B**] and whole rays [**see****Supporting Information—Fig. S3C**]. Given that percent starch did not differ by stem location [**see****Supporting Information—Fig. S3**], data were averaged across stem locations for visualization and analysis in Fig. 3. Thus, to evaluate the depletion of starch in the stem, we used a nested one-way ANOVA with stem location treated as technical replicates for each plant (treatment:stem) to analyse percent starch in the xylem ray parenchyma, phloem ray parenchyma and whole rays among treatments, respectively (Fig. 3). For significant ANOVAs, differences between pairs of means were evaluated with Tukey’s HSD, *α* = 0.05, and denoted in each figure by lowercase letters.

## Results

### Physiological traits decline over time in drought groups compared to the well-watered control group

Physiological and environmental parameters were monitored before and after the start of the experimental drought. Our soil moisture measurements support our imposed treatment groups, with soil water content remaining high throughout the experiment for the well-watered control group (~40 % volumetric water content) and declining over time for each drought group (Fig. 1A). At each timepoint prior to the start of the experimental drought, soil moisture was not significantly different between the control and drought groups (all *P* ≥ 0.09; Fig. 1A). After the start of the experimental drought, soil moisture was significantly lower in the drought groups than the well-watered control group at each timepoint (all *P* < 0.0001); for example, after 2 weeks, soil moisture was nearly five times lower in the drought groups. The experimental dry down was further confirmed by stem water potential data described at the end of this section.

Each drought group exhibited a drastic decline in photosynthetic rates throughout the experiment (Fig. 1B). By week 2 of the experimental drought, net photosynthetic rate declined to near or below zero in the 2-week, 4-week and 6-week drought groups for the remainder of the experiment. From then on, photosynthetic rate was higher in the well-watered control group than the drought groups (all pairwise comparisons, *P* < 0.01), and photosynthetic rate did not differ between the drought groups (all pairwise comparisons, *P* ≥ 0.69).

Over the course of the experiment, *F*_v_/*F*_m_ values were fairly constant and within a typical, healthy range (Björkman and Demmig 1987) in the well-watered control group (0.76 ± 0.03, mean ± SD), whereas *F*_v_/*F*_m_ declined for each drought group, indicating that water stress impacted Photosystem II in these plants (Fig. 1C). At each time point, *F*_v_/*F*_m_ was not significantly different between treatment groups (all *P* ≥ 0.13) until week 3 of the experimental drought (*F*_2, 6_ = 10.15, *P* < 0.001; Fig. 1C). Starting in week 3, *F*_v_/*F*_m_ was 7 % lower in the 6-week drought group compared to the well-watered control group (pairwise comparison, *P* = 0.03). Photosynthetic rate and *F*_v_/*F*_m_ could not be measured in the 6-week drought group beyond week 4 because the plants no longer had healthy leaves for measurement [**see****Supporting Information**—**Fig. S1**].

**Figure 2.**
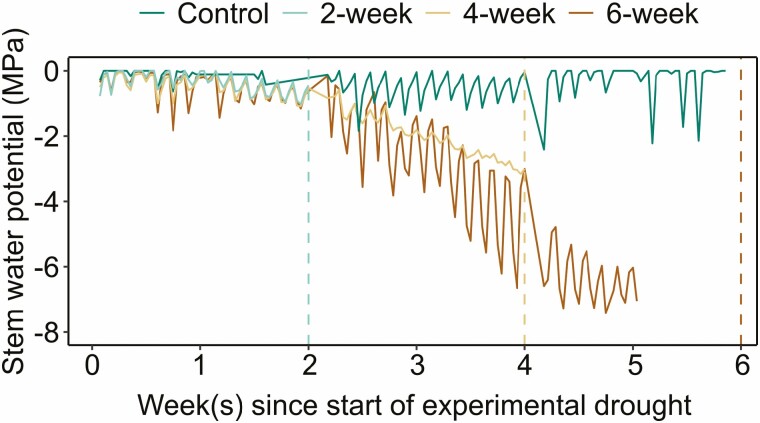
Stem water potential (in MPa) measured using ICT stem psychrometers throughout the experimental drought for four treatment groups. Vertical dashed line indicates point of harvest for each treatment, coloured by treatment. The 2-week drought group was measured for 2 weeks and then harvested, the 4-week drought group was measured for 4 weeks and then harvested, and the 6-week drought group and well-watered control group were measured for 6 weeks and then harvested. The lack of data for the 6-week drought group between weeks 5 and 6 was due to a datalogger malfunction. Data shown are daily measurements at 0:00, 6:00, 12:00 and 18:00. Hourly measurements for each treatment are displayed in **Supporting Information—Fig. S7**.

Prior to the experimental drought, weekly mid-day stem water potential, measured with the pressure chamber, showed little variation between individual plants [**see****Supporting Information—Fig. S6**]. As the experimental drought progressed, the stem water potential, measured by psychrometers, declined in the drought groups (Fig. 2). Over the course of each treatment, the mean stem water potential was −0.305 ± 0.02 MPa for the well-watered control group, −0.336 ± 0.02 MPa for the 2-week drought group, −1.23 ± 0.04 MPa for the 4-week drought group and –2.31 ± 0.08 MPa for the 6-week drought group. The lowest stem water potential, –7.42 MPa, was recorded for a plant in the 6-week drought group. Due to a datalogger malfunction, stem water potentials were not recorded for the final week of the experiment and explains the lack of data between weeks 5 and 6 for the 6-week drought group in Fig. 2. Hourly stem water potentials during the experimental drought for each treatment are provided in **Supporting Information—Fig. S7**.

### Starch depletion occurs to a greater extent in phloem ray parenchyma than xylem ray parenchyma in the stem

**Figure 3.**
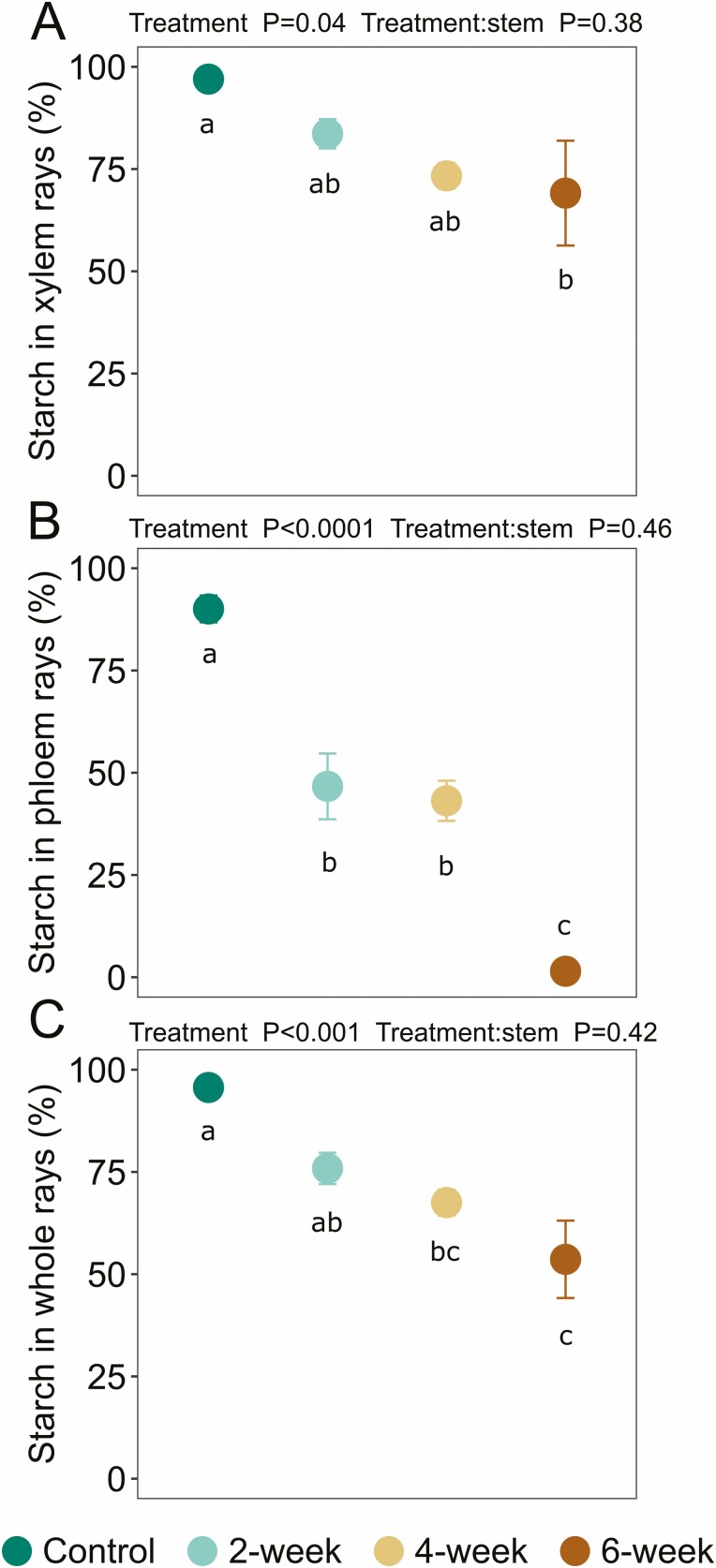
Iodine-stained starch (%) in (A) xylem ray parenchyma, (B) phloem ray parenchyma and (C) whole rays following sequential harvesting. Data were averaged over two stem locations, 10 and 50 % of the total plant length above the soil surface. The 2-week drought group underwent drought for 2 weeks and then was harvested and stained for starch, and so on for each drought group. The well-watered control group was maintained under well-watered conditions for 6 weeks and then was harvested and stained for starch. Error bars denote ± SE of the mean. In some cases, error bars are smaller than the point. Statistical results displayed above plots are from nested one-way ANOVA testing with stem location treated as technical replicates per plant (treatment:stem) and lowercase letters within the plots indicate whether treatments significantly differed from each other based on Tukey’s honest significant difference (HSD) at *α* = 0.05. Data for each stem location are provided in **Supporting Information—Fig. S3**. Data are provided in **Supporting Information—Table S1**.

**Figure 4.**
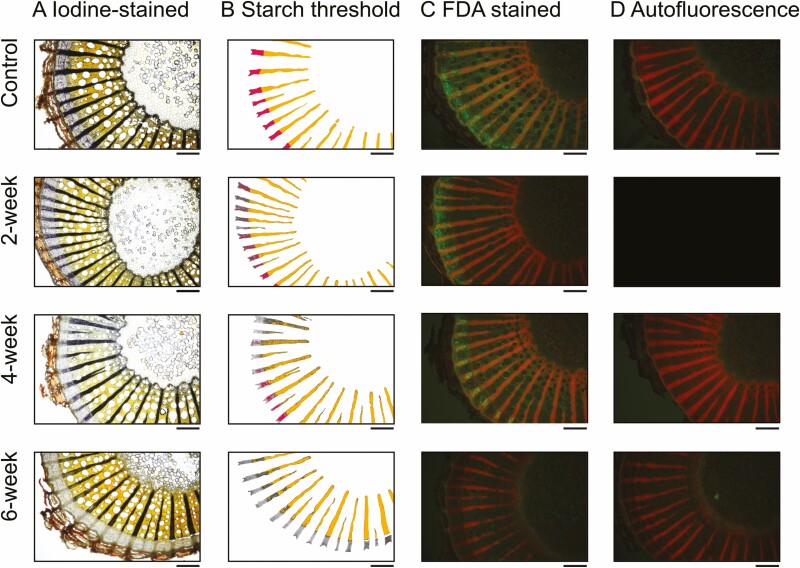
Visualization of (A) starch content and (C) metabolically active/viable cells in the stem of a representative plant from each treatment group throughout drought. Iodine-stained starch was visualized with compound light microscopy and then iodine-stained starch was identified in the xylem (orange) and phloem (pink) ray parenchyma via thresholding in ImageJ in (B). Quantification of this starch thresholding as percent starch for all plants is displayed in Fig. 3. Additionally, fluorescent living cells following FDA staining were visualized with fluorescent microscopy in (C) and autofluorescence was visualized with water in (D). Viable cells take up the stain and emit green in (C). Black box indicates missing image. Images displayed are from stem samples collected at 10 % above the soil and are from one plant per treatment (plant IDs 007, 012, 008, 005, respectively). Images for all plants are displayed in **Supporting Information—Fig. S8**. Scale bar = 0.5 mm.

The percentage of starch in the xylem and phloem ray parenchyma is displayed in Fig. 3 and these data were derived from analysis of iodine-stained images in Fig. 4. Overall, we found a steep decline in starch content in both xylem and phloem ray parenchyma over the 6-week experimental drought (Fig. 3). However, while iodine-stained starch in the xylem ray parenchyma declined by nearly 30 % between the control and 6-week drought group (pairwise comparison, *P* = 0.04); Figs 3A and 4), we saw the greatest decline in the phloem ray parenchyma with a 98 % loss of starch by week 6 (pairwise comparison, *P* < 0.0001; Figs 3B and 4).

More specifically, we observed that xylem ray parenchyma remained 97 ± 0.5 % (mean ± SE) full of starch in the well-watered control group. By week 2, starch in the xylem ray parenchyma had decreased to 83.6 ± 3.6 %, by week 4 to 73.3 ± 3.1 %, and by week 6 to 69.1 ± 12.8 % (Figs 3A and 4). Thus, starch declined by nearly 30 % in the xylem ray parenchyma following withholding water for 6 weeks. In contrast, we observed that phloem ray parenchyma remained 90.1 ± 3.3 % full of starch in the well-watered control group. By week 2, starch in the phloem ray parenchyma had decreased to 46.7 ± 8.0 %, by week 4 to 43.1 ± 4.9 %, and by week 6 to 1.5 ± 0.9 % (Figs 3B and 4). Thus, starch declined by nearly 100 % in the phloem ray parenchyma following withholding water for 6 weeks. Overall, while starch remained in the xylem ray parenchyma, the phloem ray parenchyma were nearly fully depleted of starch over the course of the experimental drought.

### Starch depletion is evident with iodine staining, enzymatic digestion and X-ray microCT

**Figure 5.**
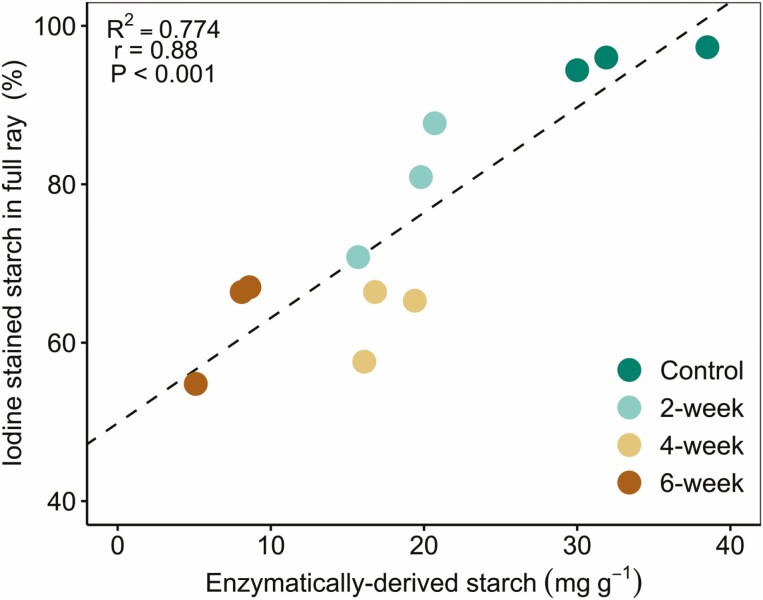
Relationship between iodine-stained starch (in %) and enzymatically derived starch concentrations (in mg g^−1^) in the stem for individual plants coloured by treatment. Samples for analyses were collected at 10 % of the total plant length above the soil surface. Strength of association was evaluated using Pearson’s correlation, *α* = 0.05.

**Figure 6.**
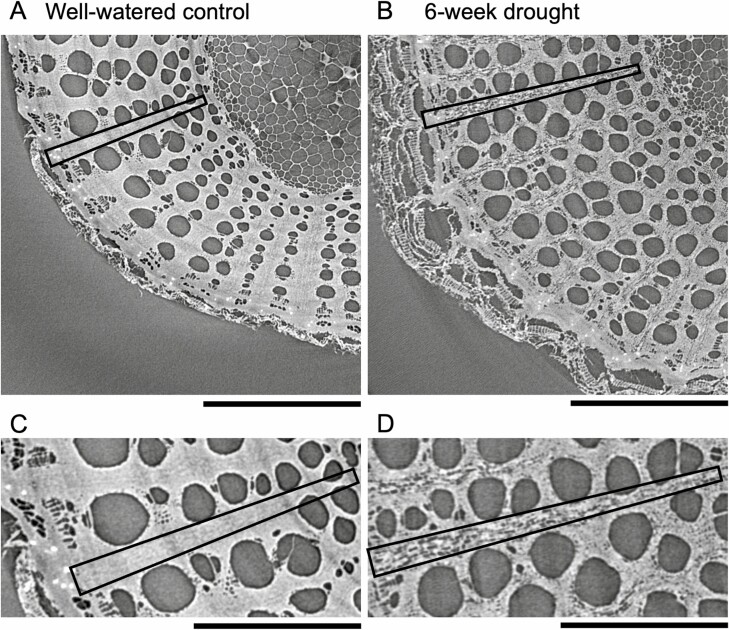
Visualization of starch in the xylem ray parenchyma of the stem in a representative (A, C) well-watered control group plant and (B, D) 6-week drought group plant using X-ray microcomputed tomography (microCT) imaging. In each stem, a single ray is outlined with a box to illustrate that in A and C the ray parenchyma is filled with starch granules and appears bright grey in colour, whereas in B and D the dark grey and black pixels within the ray parenchyma show that the ray is depleted of starch; C and D are zoomed in on the same rays outlined in A and B to show more detail. Since the stems were dried prior to microCT imaging, other tissues in the stem are no longer water-filled and thus appear dark grey (e.g. vessels and pith). Usually water-filled regions appear light grey, so drying samples improves the ability to distinguish starch from other structures. Plants imaged were plant IDs 002 and 006, respectively, and stem samples for microCT imaging were collected at 10 % of the total plant length above the soil surface. Scale bar in A and B = 1 mm, scale bar in C and D = 0.5 mm.

Iodine staining allowed us to quantify (Fig. 3) and visualize (Fig. 4) starch depletion in the phloem ray parenchyma and xylem ray parenchyma, as reported above. We also measured starch concentrations enzymatically to confirm our findings; there was a strong correlation between enzymatically derived starch and iodine-stained starch (Fig. 5); correlation between these methods has been reported previously (Fanton *et al.* 2022), but given that our study focused on a different species and abiotic stress, we independently confirmed correlation between these methods. Further, microCT imaging was more recently used to visualize starch depletion in grapevine stems in response to shade (Earles *et al.* 2018), and we also used this as a qualitative tool to support our findings about starch dynamics in the stem in response to drought. However, we hope these results will inspire future studies to use microCT imaging in a quantitative manner to assess starch dynamics in plants. Although the individual phloem cells were not visible in the microCT images, the xylem ray parenchyma were full of starch in a representative image from the well-watered control group, with rays appearing bright grey in colour, where pixel brightness is a function of X-ray attenuation and molecular density (Fig. 6A). In contrast, starch depletion was evident in the xylem ray parenchyma—appearing as dark grey and black pixels because of the absence of starch granules—in a representative image from the 6-week drought group at the end of the experimental drought (Fig. 6B). MicroCT images for all experimental groups are provided in **Supporting Information—Fig. S4**.

### The extent and timing of cell death differed between xylem and phloem under drought

FDA staining allowed us to qualitatively and quantitatively assess living regions of the xylem and phloem throughout the drought experiment, with viable/metabolically active cells absorbing the stain and emitting green fluorescence. A representative image for each treatment is displayed in Fig. 4C, but images for all plants are provided in **Supporting Information—Fig. S8**. Qualitatively, viable cells fluoresced in the xylem and phloem in the well-watered control group, but with a much higher concentration of metabolically active cells in the phloem and cambium regions compared to the xylem (control; Fig. 4C). Cells showing fluorescence in the xylem were most common in vessel-associated parenchyma flanking the vessel walls and in regions of cellular development adjacent to the vascular cambium. We found few metabolically active cells in the xylem ray parenchyma, but autofluorescence in the rays made it difficult to detect the FDA fluorescence signal in this region. After 6 weeks of drought, we found almost no viable cells in the phloem, and a few small populations of viable vasicentric parenchyma in the xylem (6-week, Fig. 4C).

Quantitatively, the FDA fluorescence intensity decreased by 45 % in the xylem after 2 weeks of drought (not statistically significant) and then did not significantly change [**see****Supporting Information—Fig. S5A**]. This aligns with our qualitative assessment of FDA images in which we observed metabolically active cells in the xylem throughout the drought. In contrast, the FDA fluorescence intensity in the phloem declined as the drought progressed, decreasing nearly 96 % between the control and 6-week drought group (pairwise comparison, *P* < 0.0001; **Supporting Information—Fig. S5B**); this agrees with our qualitative observation that there were almost no viable cells in the phloem after 6 weeks of drought.

## Discussion

### Physiological changes and declines in starch in the stem in response to drought

During drought, C-based processes directly related to NSC production and transport can become impaired, forcing plants to rely on their previously stored NSC reserves to buffer against C starvation and osmodysregulation. Recent work showed that NSC reserves provide resilience to plants during drought conditions (Adams *et al.* 2013; O’ Brien *et al.* 2014; Sevanto *et al.* 2014). Here, we observed a significant depletion of starch in *V. riparia* grapevine stems exposed to up to 6 weeks of experimental drought. Our results corroborate findings that show drought-induced NSC depletion in *V. vinifera* leaves, petioles and stems across different cultivars (Falchi *et al.* 2020; Tombesi *et al.* 2022; Vuerich *et al.* 2023), yet are in contrast with other findings showing no change in stem starch concentration (Vuerich *et al.* 2023) or drought-induced increases in stem starch (Vuerich *et al.* 2021) across other *V. vinifera* cultivars; these mixed findings further highlight the myriad of factors (e.g. organ, ontogeny, species/cultivar, legacy effects, etc.) that can influence NSC response (Adams *et al.* 2017).

Our data show that net photosynthetic rates declined to the point that no new C was being assimilated by the plants, which caused them to shift to previously fixed C to support respiration and other critical physiological processes like turgor maintenance, growth and defense. This switch to existing C reserves occurred by the second week of drought (i.e. no photosynthesis, Fig. 1B). During the first 2 weeks, the experimental drought conditions likely initially induced stomatal closure (Choat *et al.* 2018), leading to measured declines in photosynthesis, chlorophyll fluorescence and stem water potential (Figs 1 and 2). During this same period, we also observed starch begin to decline in the xylem and phloem ray parenchyma, a process that manifests when starch-degrading enzymes are activated and begin to break down starch into soluble sugars to support the maintenance of metabolism and osmoregulation (Basu *et al.* 2007; Krasensky and Jonak 2012; Thalmann and Santelia 2017; Guo *et al.* 2020). Recent work also suggests that stem NSCs are important for reducing xylem vulnerability to embolism and improving drought outcome (Tomasella *et al.* 2021; Natale *et al.* 2023).

### Xylem and phloem responses to drought are distinct

Notably, our findings show distinct differences in starch depletion and metabolic activity within the xylem and phloem ray parenchyma during simulated drought (Figs 3 and 4). While nearly all of the starch in the phloem was consumed by the end of the drought with only 1.5 ± 0.9 % remaining, starch was consumed in the xylem to a lesser extent with 69.1 ± 12.8 % remaining. And while the phloem was the first to show loss of cell viability, some metabolically active parenchyma cells remained in the xylem at the end of the experiment. These spatial dynamics suggest both higher populations of metabolically active cells but also commensurately potentially higher respiration rates in the phloem and adjacent cambium that rapidly depleted local NSC pools and led to cell death, while the larger cross-sectional area of the xylem ray parenchyma with more NSC storage and lower metabolically active cell populations depleted starch at a slower pace and maintained cell viability in this region. Further, starch depletion could have been used as a way to increase the soluble sugar fraction to buffer photosynthesis limitations under drought (He *et al.* 2020; Long and Adams 2023). While we did not measure soluble sugars in our study, a quantitative evaluation of starch and soluble sugars in both the xylem and phloem would help resolve the underlying mechanisms leading to drought-induced mortality in future studies.

Similar to our findings in grapevines, NSCs are often not fully used up during drought in trees (Adams *et al.* 2017). The unconsumed starch in the xylem ray parenchyma may indicate that the source-sink relationship between the xylem and the phloem may not have been strong enough to stimulate the movement of NSCs across the cambium and into the phloem, or the resistance of that pathway was significantly higher. Since the phloem exchanges NSCs with surrounding tissues (Epron *et al.* 2015) and redistributes NSCs to starving tissues and organs under typical conditions, its loss of function can impact access to NSC reserves (Sevanto 2014; Sevanto *et al.* 2014). Loss of phloem function has been proposed to occur as the build-up of soluble sugars needed for osmotic adjustment during dehydration may increase phloem sap viscosity and halt transport in the phloem, although collapse may occur prior to this (Hölttä *et al.* 2006, 2009; Sevanto 2014; Salmon *et al.* 2018); this further highlights the utility of measuring soluble sugars in addition to starch in future studies. A recent study investigating the fire-induced mortality of conifer saplings found that phloem death, rather than xylem hydraulic failure, was a major driver of plant death (Partelli-Feltrin *et al.* 2022). However, despite the phloem’s importance, we lack empirical evidence about phloem responses to drought due to the challenge of measuring and characterizing its properties (Savage *et al.* 2016; Sevanto 2018).

Given the cessation of photosynthesis along with extensive starch depletion and cell death in the phloem at the conclusion of our study, we presume loss of phloem function and also have evidence for hydraulic failure in the droughted plants. Lamarque *et al.* 2023) showed that the mean value of xylem pressure inducing 50 and 88 % loss of hydraulic conductivity in *V. riparia* stems was –3.05 ± 0.14 and –4.36 ± 0.14 MPa, respectively. Plants in our study had xylem water potentials between −3 and −5 MPa by weeks 3–4, suggesting that hydraulic failure could have occurred. It is then possible that starch may not have been fully consumed in the stem xylem because it could not be metabolized, could not be transported to organs in need (e.g. roots) (Sala *et al.* 2010), or such sink organs were already dead. There is evidence from trees, however, that they can survive after complete vascular cambium and phloem damage using only stored NSCs in a given organ. For instance, when root respiration was decoupled from canopy assimilation, NSCs were transferred within the root system to maintain respiration for over a year (Aubrey and Teskey 2018).

This finding elicits another possibility for the remaining starch in the xylem ray parenchyma—to aid in persistence and recovery. Investing resources into maintenance and recovery processes in anticipation that rainfall/irrigation will eventually return could be a strategy employed by plants—with stored starch serving as a source of soluble sugars to be used as osmoticum for refilling and as an energy source for regrowth and respiration (Secchi and Zwieniecki 2011; Brodersen and McElrone 2013; Tomasella *et al.* 2017). Previous studies have shown that recovery of NSCs in *V. vinifera* following short-term water stress (6–10 days) depended on the cultivar and the type of NSC; in some cases, soluble NSCs recovered to pre-drought levels after rewatering (Vuerich *et al.* 2023), while starch did not recover in petiole tissue following rewatering (Falchi *et al.* 2020). While we did not assess whether our plants were dead beyond quantifying cell viability in the phloem and xylem, our experimental drought was much longer than the above studies, and we believe the plants were indeed dead; however, if the root system was still living, resprouting may have occurred following rewatering. Regardless, whether the functionality of the stem xylem ray parenchyma would persist and its starch could be accessed to support the regrowth of the xylem (Trugman *et al.* 2018) or phloem following rewatering after drought, or if the recovery of the phloem is even plausible beyond a critical point, is unknown.

### Conclusion

We have assessed the impacts of drought on the C physiology of *V. riparia* stems over the course of a 6-week experimental drought. We showed that starch depletion occurred in the stems of droughted plants and generally continued over time; depletion in the ray parenchyma was quantified after visualization of starch with iodine staining and was further supported by results from enzymatic starch digestion and qualitative X-ray microCT imaging. Iodine staining has been used to identify starch in previous studies (Atkinson *et al.* 2012; Fanton *et al.* 2022) and we took advantage of this tool to characterize and compare starch dynamics along with cell viability in the xylem and phloem throughout drought. Notably, we observed differences in the timing and extent of starch depletion and cell viability between the xylem and the phloem, with the phloem experiencing near-complete depletion and death. Therefore, our work provides insight into the contributions of within-organ C dynamics to abiotic stress response at the whole-plant level, and highlights the importance of considering both the xylem and the phloem together in future NSC-stress studies.

## Supporting Information

The following additional information is available in the online version of this article –

**Figure S1.** Example of plant (marked with red asterisk; ID 003) from the 6-week drought group at (A) 4 weeks and (B) 5 weeks after the start of the experimental drought. Photosynthesis and chlorophyll fluorescence measurements were not measured for droughted-plants in the final two weeks (weeks 5 and 6) of the experiment because they no longer had healthy leaves.

**Figure S2.** Comparison of stem water potential (MPa) measured by each ICT psychrometer (*n* = 4) and a pressure chamber in the afternoon of August 25, 2020 on the day before the start of the experimental drought. This comparison was conducted to ensure that the psychrometer measurements were giving valid stem water potentials. The pressure chamber was used to measure stem water potentials prior to the start of the experimental drought [**see****Supporting Information—Fig. S6**], and the psychrometers were used to monitor stem water potentials throughout the experimental drought (see Fig. 2, **Supporting Information—Fig. S7**).

**Figure S3**. Iodine-stained starch (%) in (A) xylem ray parenchyma, (B) phloem ray parenchyma, and (C) whole rays at two stem locations, 10 % (left column) and 50 % (right column) of the total plant length above the soil surface following sequential harvesting. Error bars denote ± 1 SD of the mean. Statistical results displayed above plots are from two-way ANOVA testing (treatment × stem location). Data are provided in **Supporting Information—Table S1**. Since starch did not differ by stem location, data were averaged across stem locations for visualization and analysis in Fig. 3.

**Figure S4**. MicroCT images of stems from the well-watered control group (A-C), the 2-week drought group (D-F), the 4-week drought group (G-I), and the 6-week drought group (J-L). Ray parenchyma are full of starch granules when appearing bright gray in color, and depleted when appearing dark gray or black in color. All stems were collected at 10 % above the soil. Plant IDs are listed in white text in the lower left corner of each image. Scale bar = 1 mm.

**Figure S5.** Fluorescence intensity in the (A) xylem and (B) phloem of stems across drought treatment groups. Fluorescence intensity was quantified using the integrated density measurement in FIJI on FDA-stained images of the stem at 10% and 50 % of the total plant length above the soil surface. Error bars denote ± SE of the mean. Statistical results displayed above plots are from one-way nested ANOVA testing with stem location treated as technical replicates per plant (treatment:stem) and lowercase letters within the plots indicate whether treatments significantly differed from each other based on Tukey’s honest significant difference (HSD) at *α* = 0.05.

**Figure S6.** Weekly stem water potential (MPa) measured using a Scholander pressure chamber for each individual plant prior to the start of the experimental drought. Black points represent individual plants (*n* = 12), while red points represent the mean. Three weeks prior to the start of the experimental drought, water potentials were measured on August 7, 2020 between approximately 16:00-16:30, whereas water potentials were measured earlier between approximately 13:30-14:30 in the two weeks that followed (on August 14, 2020 and August 21, 2020).

**Figure S7**. Hourly stem water potential (in MPa) measured using ICT stem psychrometers for the (A) well-watered control, (B) 2-week drought group, (C) 4-week drought, and (D) 6-week drought groups.

**Figure S8.** Visualization of (A) starch content and (C) metabolically active cells in the stem of each plant by treatment following drought. Iodine-stained starch was visualized with compound light microscopy and then iodine-stained starch was identified in the xylem (orange) and phloem (pink) ray parenchyma via thresholding in ImageJ in (B). Quantification of this starch thresholding as percent starch for all plants is displayed in Fig. 3. Additionally, fluorescent living cells following FDA staining were visualized with fluorescent microscopy in (C) and autofluorescence was visualized with water in (D). Black box indicates missing image. Images shown are for stem samples at 10 % above the soil, but data from 50 % above the soil was also collected. Scale bar = 0.5 mm

**Table S1** Percent of starch in the xylem ray parenchyma, phloem ray parenchyma, and whole rays quantified using thresholding in ImageJ as well as starch concentration from enzymatic digestion for each individual plant. Percent starch was quantified at two stem locations, 10% and 50 % above the soil. Enzymatically-derived starch concentrations were quantified at 10 % above the soil.

plad062_suppl_Supplementary_Material

## Data Availability

The data underlying this article are available in its online supplementary material and any additional reasonable requests beyond this should be directed to the corresponding author.

## Notes

**Form & Function. Chief Editor:** Kate McCulloh
