# Supplementary material for: Starch depletion in the xylem and phloem ray parenchyma of grapevine stems under drought
Source: AoB Plants. 2023 Aug 30;15(5):plad062. doi: 10.1093/aobpla/plad062 (PMC10601394; doi:10.1093/aobpla/plad062)
Supplement: plad062_suppl_Supplementary_Material [file plad062_suppl_supplementary_material.pdf]

**Supporting Information**

A) 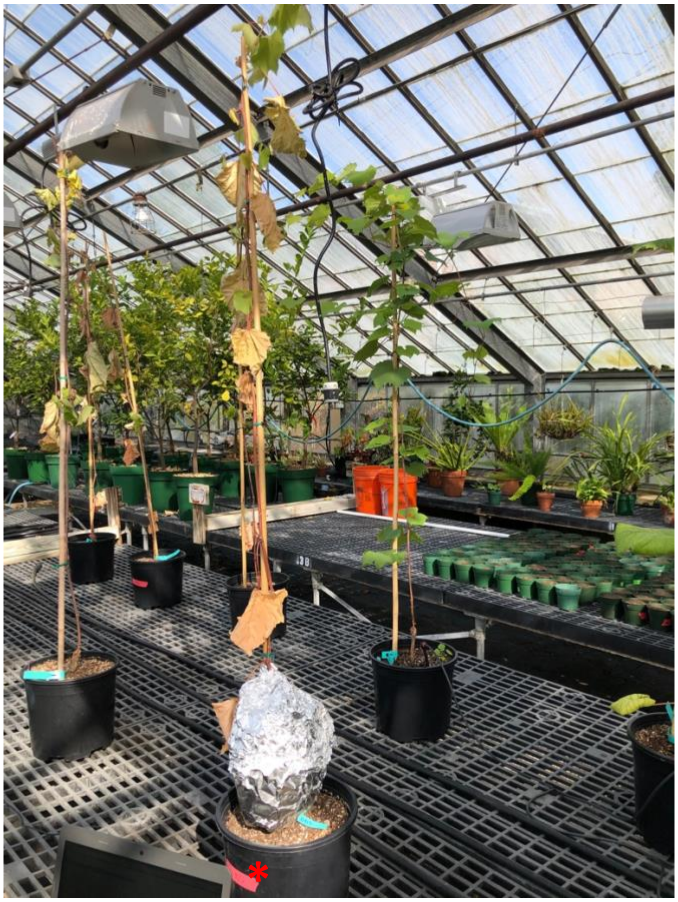

B) 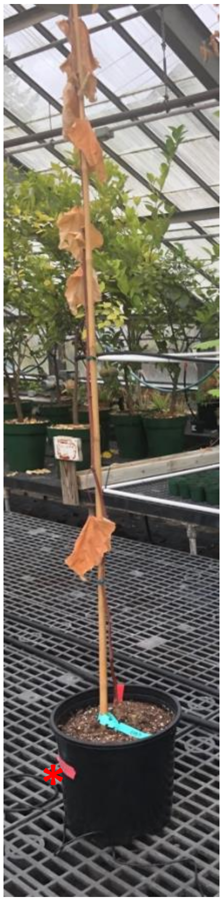

**Figure S1.** Example of plant (marked with red asterisk; ID 003) from the 6-week drought group at (A) 4 weeks and (B) 5 weeks after the start of the experimental drought. Photosynthesis and chlorophyll fluorescence measurements were not measured for droughted-plants in the final two weeks (weeks 5 and 6) of the experiment because they no longer had healthy leaves.

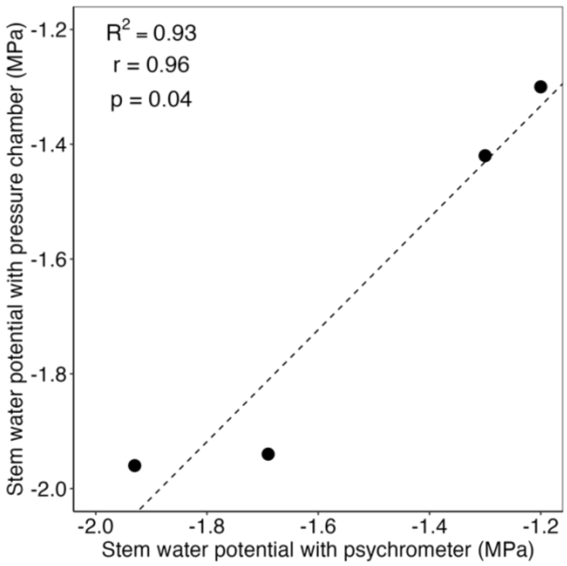


**Figure S2.** Comparison of stem water potential (MPa) measured by each ICT psychrometer (n=4) and a pressure chamber in the afternoon of August 25, 2020 on the day before the start of the experimental drought. This comparison was conducted to ensure that the psychrometer measurements were giving valid stem water potentials. The pressure chamber was used to measure stem water potentials prior to the start of the experimental drought (see Fig. S6), and the psychrometers were used to monitor stem water potentials throughout the experimental drought (see Figs. 2, S7).

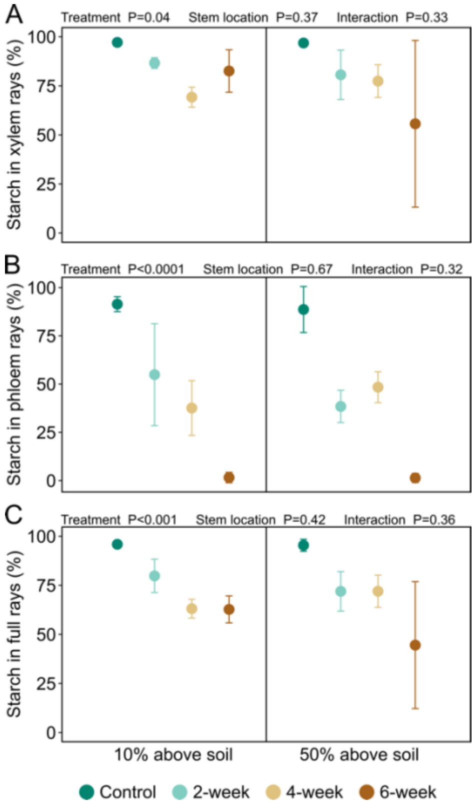


**Figure S3**. Iodine-stained starch (%) in (A) xylem ray parenchyma, (B) phloem ray parenchyma, and (C) whole rays at two stem locations, 10% (left column) and 50% (right column) of the total plant length above the soil surface following sequential harvesting. Error bars denote ± 1 SD of the mean. Statistical results displayed above plots are from two-way ANOVA testing (treatment x stem location). Data are provided in Table S1. Since starch did not differ by stem location, data were averaged across stem locations for visualization and analysis in Fig. 3.

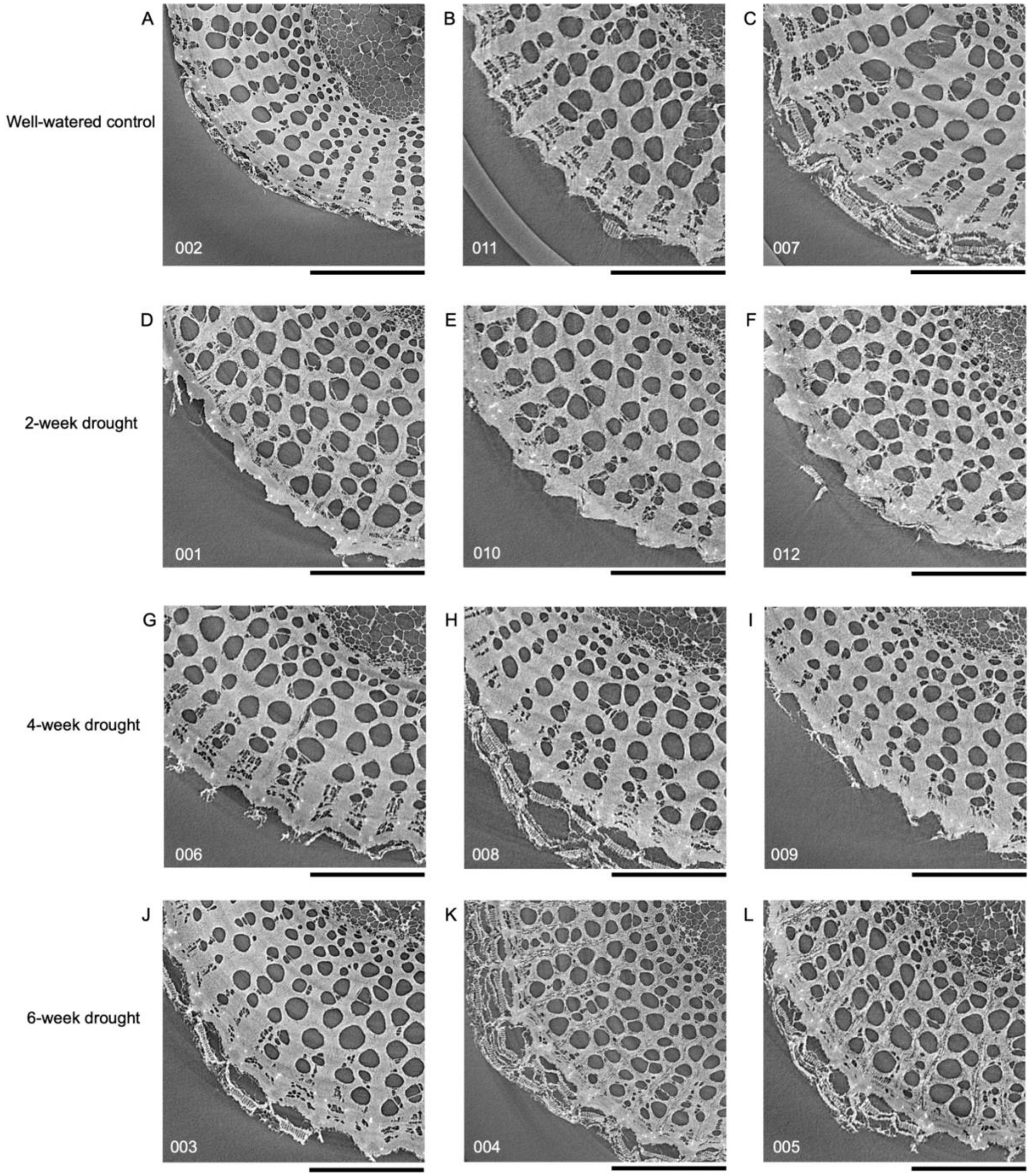


**Figure S4**. MicroCT images of stems from the well-watered control group (A-C), the 2-week drought group (D-F), the 4-week drought group (G-I), and the 6-week drought group (J-L). Ray parenchyma are full of starch granules when appearing bright gray in color, and depleted when appearing dark gray or black in color. All stems were collected at 10% above the soil. Plant IDs are listed in white text in the lower left corner of each image. Scale bar = 1 mm.

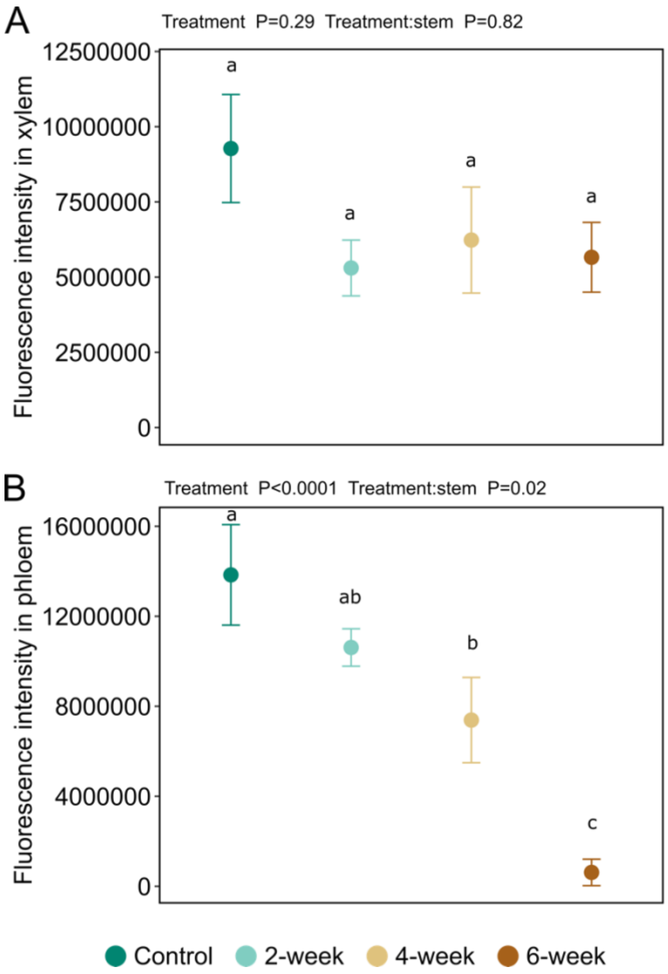


**Figure S5.** Fluorescence intensity in the (A) xylem and (B) phloem of stems across drought treatment groups. Fluorescence intensity was quantified using the integrated density measurement in FIJI on FDA-stained images of the stem at 10% and 50% of the total plant length above the soil surface. Error bars denote ± SE of the mean. Statistical results displayed above plots are from one-way nested ANOVA testing with stem location treated as technical replicates per plant (treatment:stem) and lowercase letters within the plots indicate whether treatments significantly differed from each other based on Tukey's honest significant difference (HSD) at $\alpha = 0.05$

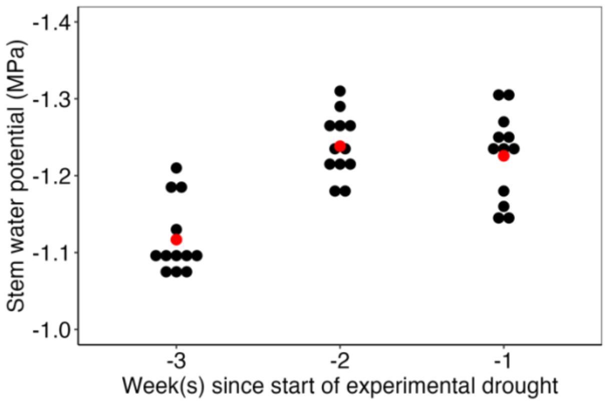


**Figure S6.** Weekly stem water potential (MPa) measured using a Scholander pressure chamber for each individual plant prior to the start of the experimental drought. Black points represent individual plants (n=12), while red points represent the mean. Three weeks prior to the start of the experimental drought, water potentials were measured on August 7, 2020 between approximately 16:00-16:30, whereas water potentials were measured earlier between approximately 13:30-14:30 in the two weeks that followed (on August 14, 2020 and August 21, 2020).

(A)

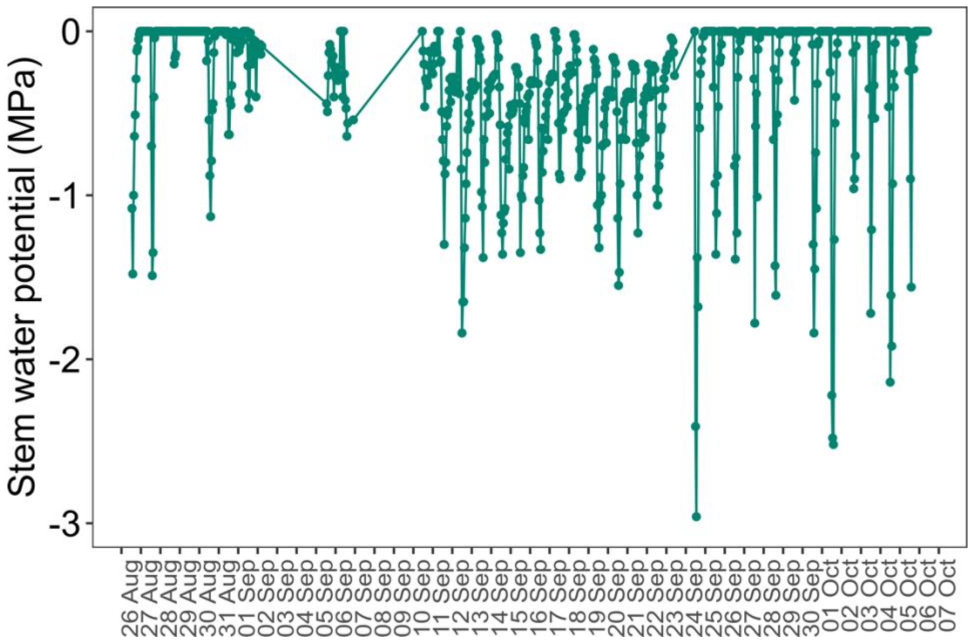

Stem water potential (MPa)
0
-1
-2
-3
26 Aug
27 Aug
28 Aug
29 Aug
30 Aug
31 Aug
01 Sep
02 Sep
03 Sep
04 Sep
05 Sep
06 Sep
07 Sep
08 Sep
09 Sep
10 Sep
11 Sep
12 Sep
13 Sep
14 Sep
15 Sep
16 Sep
17 Sep
18 Sep
19 Sep
20 Sep
21 Sep
22 Sep
23 Sep
24 Sep
25 Sep
26 Sep
27 Sep
28 Sep
29 Sep
30 Sep
01 Oct
02 Oct
03 Oct
04 Oct
05 Oct
06 Oct
07 Oct


(B)

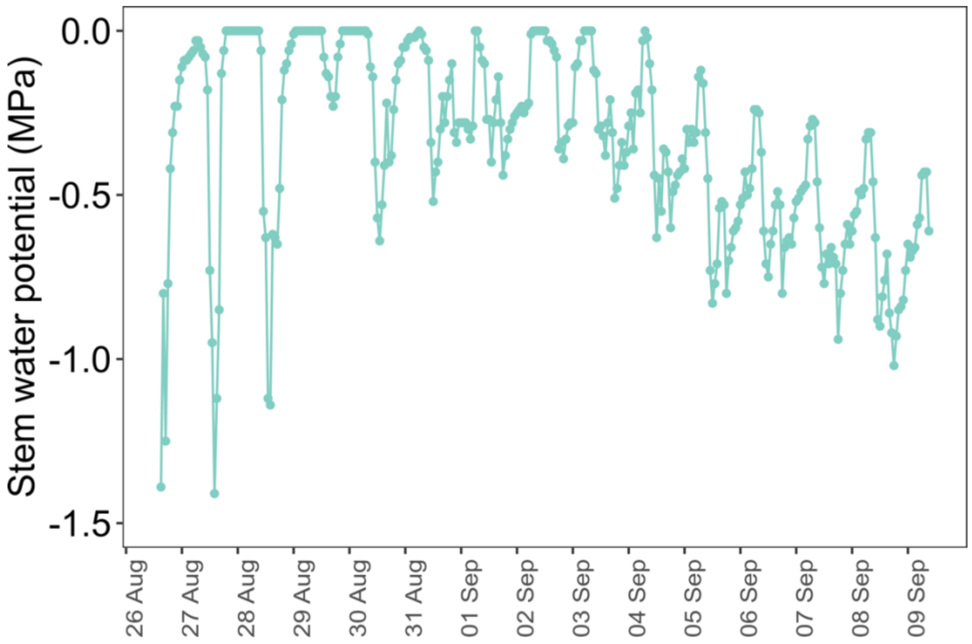

Stem water potential (MPa)
0.0
-0.5
-1.0
-1.5
26 Aug
27 Aug
28 Aug
29 Aug
30 Aug
31 Aug
01 Sep
02 Sep
03 Sep
04 Sep
05 Sep
06 Sep
07 Sep
08 Sep
09 Sep

(C)

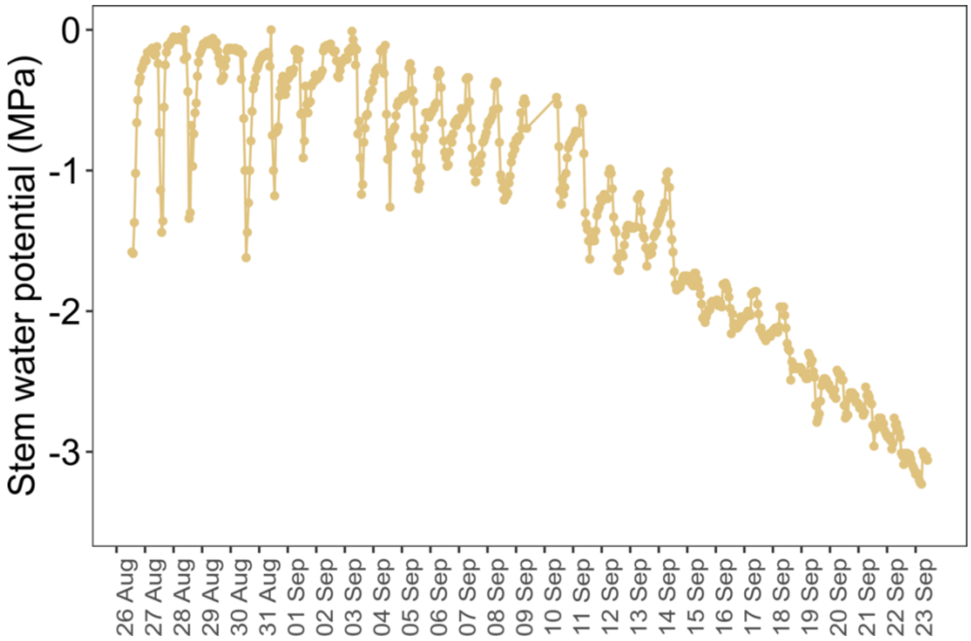


(D)

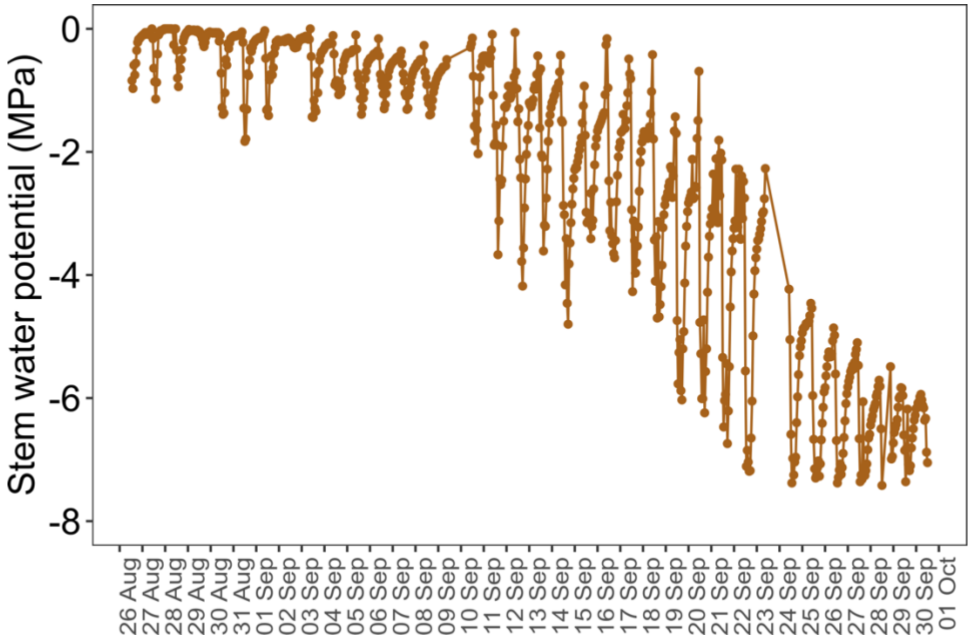


**Figure S7**. Hourly stem water potential (in MPa) measured using ICT stem psychrometers for the (A) well-watered control, (B) 2-week drought group, (C) 4-week drought, and (D) 6-week drought groups.

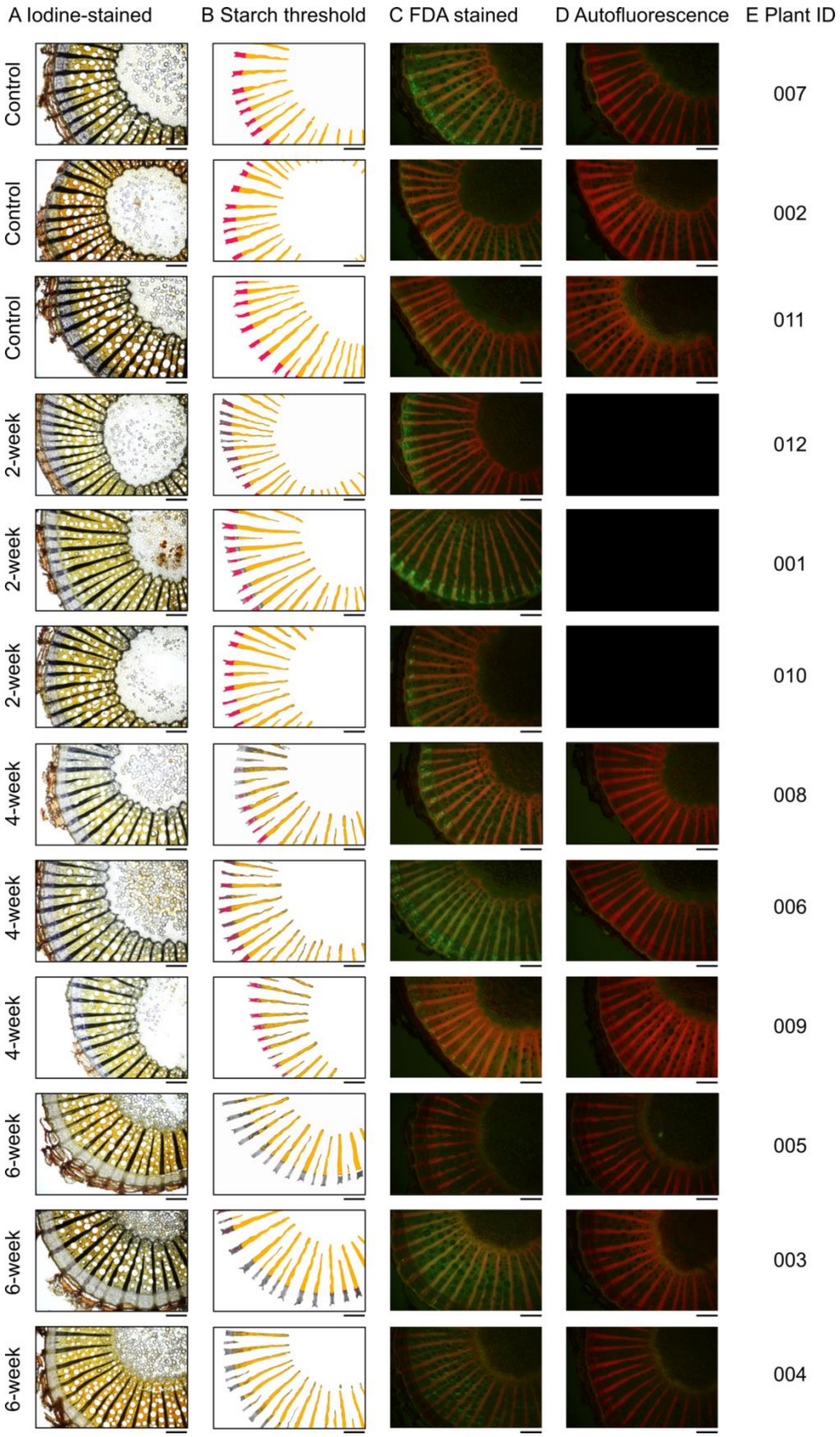
A Iodine-stained
B Starch threshold
C FDA stained
D Autofluorescence
E Plant ID
Control
Control
Control
2-week
2-week
2-week
4-week
4-week
4-week
6-week
6-week
6-week
007
002
011
012
001
010
008
006
009
005
003
004

**Figure S8.** Visualization of (A) starch content and (C) metabolically active cells in the stem of each plant by treatment following drought. Iodine-stained starch was visualized with compound light microscopy and then iodine-stained starch was identified in the xylem (orange) and phloem (pink) ray parenchyma via thresholding in ImageJ in (B). Quantification of this starch thresholding as percent starch for all plants is displayed in Fig. 3. Additionally, fluorescent living cells following FDA staining were visualized with fluorescent microscopy in (C) and autofluorescence was visualized with water in (D). Black box indicates missing image. Images shown are for stem samples at 10% above the soil, but data from 50% above the soil was also collected. Scale bar = 0.5 mm

**Table S1** Percent of starch in the xylem ray parenchyma, phloem ray parenchyma, and whole rays quantified using thresholding in ImageJ as well as starch concentration from enzymatic digestion for each individual plant. Percent starch was quantified at two stem locations, 10% and 50% above the soil. Enzymatically-derived starch concentrations were quantified at 10% above the soil.

| plant ID | stem location | treatment | starch in xylem rays (%) | starch in phloem rays (%) | starch in whole rays (%) | starch concentration (mg $g^{-1}$) |
|---|---|---|---|---|---|---|
| 003 | 50 | 6-week drought | 95.4 | 4.1 | 72.5 | NA |
| 003 | 10 | 6-week drought | 95.0 | 4.7 | 67.0 | 8.6 |
| 005 | 50 | 6-week drought | 60.7 | 0.0 | 52.0 | NA |
| 005 | 10 | 6-week drought | 75.3 | 0.0 | 54.8 | 5.1 |
| 004 | 50 | 6-week drought | 10.9 | 0.0 | 9.1 | NA |
| 004 | 10 | 6-week drought | 77.4 | 0.0 | 66.4 | 0 |
| 006 | 50 | 4-week drought | 68.2 | 39.2 | 62.5 | NA |
| 006 | 10 | 4-week drought | 74.9 | 35.6 | 66.4 | 18.8 |
| 008 | 50 | 4-week drought | 84.4 | 51.9 | 77.2 | NA |
| 008 | 10 | 4-week drought | 65.0 | 24.5 | 57.6 | 17.1 |
| 009 | 50 | 4-week drought | 79.7 | 54.1 | 76.2 | NA |
| 009 | 10 | 4-week drought | 67.8 | 53.5 | 65.1 | 19.4 |
| 001 | 50 | 2-week drought | 66.2 | 29.2 | 60.7 | NA |
| 001 | 10 | 2-week drought | 85.1 | 60.9 | 80.9 | 13.7 |
| 0010 | 50 | 2-week drought | 86.4 | 40.6 | 75.0 | NA |
| 0010 | 10 | 2-week drought | 89.8 | 77.8 | 87.7 | 19.8 |
| 0012 | 50 | 2-week drought | 89.3 | 45.5 | 80.1 | NA |
| 0012 | 10 | 2-week drought | 85.0 | 26.0 | 70.8 | 20.7 |
| 002 | 50 | control | 95.8 | 75.5 | 92.7 | NA |
| 002 | 10 | control | 97.3 | 91.0 | 96.0 | 21.9 |
| 0011 | 50 | control | 98.7 | 98.7 | 98.7 | NA |
| 0011 | 10 | control | 97.8 | 95.5 | 97.3 | 30 |
| 007 | 50 | control | 96.0 | 91.8 | 94.8 | NA |
| 007 | 10 | control | 96.3 | 87.8 | 94.4 | 38.5 |
